# Fungal Dysbiosis and Intestinal Inflammation in Children With Beta-Cell Autoimmunity

**DOI:** 10.3389/fimmu.2020.00468

**Published:** 2020-03-19

**Authors:** Jarno Honkanen, Arja Vuorela, Daniel Muthas, Laura Orivuori, Kristiina Luopajärvi, Mysore Vishakante Gowda Tejesvi, Anton Lavrinienko, Anna Maria Pirttilä, Christopher L. Fogarty, Taina Härkönen, Jorma Ilonen, Terhi Ruohtula, Mikael Knip, Janne J. Koskimäki, Outi Vaarala

**Affiliations:** ^1^Clinicum, Faculty of Medicine, University of Helsinki, Helsinki, Finland; ^2^Translational & Experimental Medicine, Early Respiratory, Inflammation and Autoimmunity, BioPharmaceuticals R&D, AstraZeneca, Gothenburg, Sweden; ^3^Children's Hospital, University of Helsinki and Helsinki University Hospital, Helsinki, Finland; ^4^Ecology and Genetics Research Unit, University of Oulu, Oulu, Finland; ^5^Department of Biological and Environmental Science, University of Jyväskylä, Jyväskylä, Finland; ^6^Research Program for Clinical and Molecular Metabolism, Faculty of Medicine, University of Helsinki, Helsinki, Finland; ^7^Immunogenetics Laboratory, University of Turku, Turku, Finland

**Keywords:** mycobiome, dysbiosis, gut, inflammation, *Candida*, *Saccharomyces*, type 1 diabetes

## Abstract

Although gut bacterial dysbiosis is recognized as a regulator of beta-cell autoimmunity, no data is available on fungal dysbiosis in the children at the risk of type 1 diabetes (T1D). We hypothesized that the co-occurrence of fungal and bacterial dysbiosis contributes to the intestinal inflammation and autoimmune destruction of insulin-producing beta-cells in T1D. Fecal and blood samples were collected from 26 children tested positive for at least one diabetes-associated autoantibody (IAA, GADA, IA-2A or ICA) and matched autoantibody-negative children with HLA-conferred susceptibility to T1D (matched for HLA-DQB1 haplotype, age, gender and early childhood nutrition). Bacterial 16S and fungal ITS2 sequencing, and analyses of the markers of intestinal inflammation, namely fecal human beta-defensin-2 (HBD2), calprotectin and secretory total IgA, were performed. Anti-Saccharomyces cerevisiae antibodies (ASCA) and circulating cytokines, IFNG, IL-17 and IL-22, were studied. After these analyses, the children were followed for development of clinical T1D (median 8 years and 8 months). Nine autoantibody positive children were diagnosed with T1D, whereas none of the autoantibody negative children developed T1D during the follow-up. Fungal dysbiosis, characterized by high abundance of fecal *Saccharomyces* and *Candida*, was found in the progressors, i.e., children with beta-cell autoimmunity who during the follow-up progressed to clinical T1D. These children showed also bacterial dysbiosis, i.e., increased Bacteroidales and Clostridiales ratio, which was, however, found also in the non-progressors, and is thus a common nominator in the children with beta-cell autoimmunity. Furthermore, the progressors showed markers of intestinal inflammation detected as increased levels of fecal HBD2 and ASCA IgG to fungal antigens. We conclude that the fungal and bacterial dysbiosis, and intestinal inflammation are associated with the development of T1D in children with beta-cell autoimmunity.

## Introduction

Type 1 diabetes (T1D) is an immune-mediated disease in which autoimmune mechanisms are considered to be responsible for the destruction of insulin-producing pancreatic beta cells. While the triggers of the disease process remain open, the development of local inflammation in the pancreatic islets and formation of autoantibodies against beta-cell antigens are early events in the development of T1D (1–4). Autoantibodies emerge against various beta-cell antigens, such as insulin, glutamate decarboxylase, islet antigen 2, and zinc transporter 8, several years before the clinical disease manifestation, and the risk of T1D correlates with the number of beta-cell autoantibodies. Other immunological aberrancies in T1D include up-regulation of IFNG and IL-17 pathways (5–9). We have previously shown that children with beta-cell autoimmunity have a decreased abundance of butyrate-producing bacteria and an increased abundance of bacteria belonging to the phylum Bacteroidetes in their gut microbiota (10, 11). Similar alterations in the bacterial community in children with beta-cell autoimmunity have been confirmed in several later studies (12–14). Intestinal inflammation has been associated with T1D as demonstrated by up-regulated expression of HLA class II molecule and cytokines IFNG, TNFA and IL-4 mRNA in jejunal biopsies (15).

The role of gut microbiota as a regulator of autoimmune diabetes is well-established in animal models of T1D, in which modulation of the microbiota affects the disease development (16). To date, the studies of the microbiome in relation to T1D have focused on the bacterial community of the gut microbiota, however, human microbiome is a complex ecosystem composed of bacteria, fungi, archaea, and viruses. Several fungal species have been identified in the human gastrointestinal tract (17, 18), representing 0.1–1.0% of the intestinal microbiota (commonly referred to as *mycobiota*). The fungal cells are outnumbered by the bacterial ones, but as eukaryotic organisms, fungi have substantially more diverse biochemical pathways than bacteria (19). Thus, when the bioactive capacity of the intestinal microbiota is considered, the role of mycobiota is of major importance with a remarkable potential to modulate host cellular functions. That said, the current knowledge of the involvement of mycobiota in the perturbations of the microbial communities and host health is limited. The role of mycobiota as a regulator of intestinal inflammation and inflammatory diseases has been emphasized by recent studies in inflammatory bowel disease, allergy, and asthma (20–22).

Moreover, the changes in the bacterial microbiota may be linked to the alterations of the mycobiota, which are likely disrupting the interkingdom interactions within the microbiome, as seen in Crohn's disease (21, 22). Indeed, intestinal mycobiota can modulate the composition of the bacterial compartment either by direct interactions with bacteria, or via the immune system of the host (18, 23).

In the current study, we analyzed the composition of the fungal and bacterial gut microbiota, as well as markers of intestinal inflammation, in a cohort of islet autoantibody positive and negative children carrying HLA-conferred genetic susceptibility to T1D. We then followed the cohort for the development of T1D for median of 8 years and 8 months. Combining the fungal and bacterial data, the children with genetic risk of T1D were grouped into three major clusters defined by the relative abundance of *Saccharomyces*, Clostridiales, and Bacteroidales (Firmicutes and Bacteroidetes phyla, respectively). An increased ratio of Bacteroidales to Clostridiales was found in autoantibody positive children while the children who during the follow-up also progressed to clinical T1D, showed high abundance of *Saccharomyces* and *Candida*, as well as signs of intestinal inflammation, i.e., increased levels of fecal HBD2 and circulating ASCA IgG. Our results indicate that dysbiosis of fungal and bacterial gut microbiota as well as intestinal inflammation are associated with the development of T1D.

## Materials and Methods

### Study Subjects

**Figure 1:**
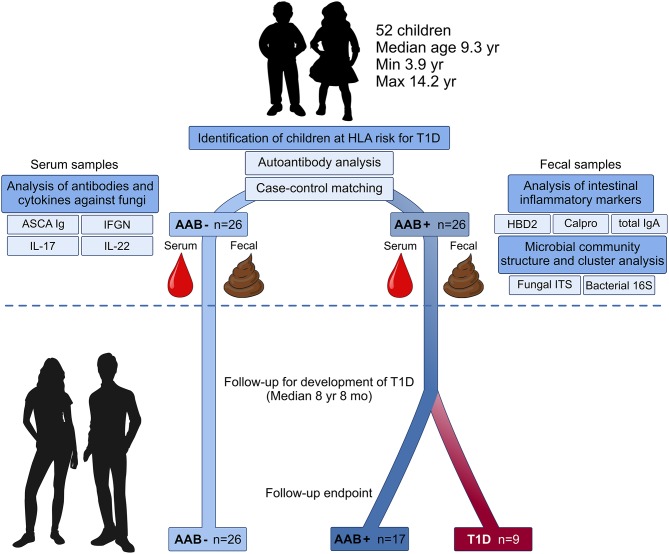
Study design: Microbiome composition, intestinal inflammation and the development of clinical type 1 diabetes (T1D) during the follow-up. Fecal and blood samples were collected from 26 children tested positive for at least one diabetes-associated autoantibody (IAA, GADA, IA-2A, or ICA) and matched autoantibody-negative children with HLA-conferred susceptibility to T1D. Case-control pairs were matched for HLA-DQB1 haplotype, age, gender, and early childhood nutrition. Bacterial 16S and fungal ITS2 sequencing and analyses of the markers of intestinal inflammation, namely HBD2, calprotectin, and secretory total IgA, were performed using fecal samples. Blood samples were analyzed for the levels of ASCA IgA/IgG and circulating cytokines IFNG, IL-17, and IL-22. After the analyses, the children were followed for development of clinical T1D (median 8 years and 8 months). During the follow-up nine autoantibody-positive children were diagnosed with T1D, whereas none of the autoantibody-negative children developed T1D.

Experimental design of the current study is presented in Figure 1. Here we collected fecal and blood samples from 52 children with HLA-conferred susceptibility to T1D (Table 1) and followed them for the development of T1D for a median of 8 years and 8 months (range 8 years and 2 months−9 years and 1 month). The children studied for fecal microbiome were recruited from the participants of the nutritional intervention studies (24–26). We identified 26 children tested positive for at least one T1D-associated autoantibody (IAA, GADA, IA-2A, or ICA) (cases), and selected autoantibody-negative healthy control children matched for age, sex, HLA-DQB1 genotype and early life nutrition. At the start of the follow-up, fecal samples were collected (between February 2009 and February 2010) using stool collection vials and immediately stored in home freezers (−20°C). The frozen samples were delivered to the study center, and the samples were stored at −80°C until processing. At the time of fecal sample collection, the study subjects did not have gastroenteritis and had not received antibiotic treatment during the past 3 months. Nine children developed T1D during the follow-up. The control children remained non-diabetic and negative for all four autoantibodies analyzed. The study was approved by the ethics committees of the participating hospitals and the families and/or the children taking part in the study gave their written informed consent.

**Table 1:** Characteristics of the study subjects. AAb+ are children positive for at least one diabetes-associated autoantibody and AAb- children are negative for beta-cell autoantibodies. The study subjects were participants in the TRIGR and FINDIA pilot studies.

**Characteristics**	**AAb+ children (*N* = 26)**	**AAb– children (*N* = 26)**
Female/male	9/17	9/17
**Age (years)**		
TRIGR pilot study	13.3 (11.7–14.2)	12.7 (11.9–13.6)
FINDIA pilot study	5.1 (4.0–6.1)	5.3 (3.9–7.0)
**HLA-DQB1 genotype**		
*02:0302	7	7
*03:02/x	12	11
*02(DQA1*05)/y	5	7
*02(DQA1*03)/y	1	0
*02(DQA1*02:01)	1	1

Data are number (N) or medians (with range). X not DQB1*02, DQB1*03:01, or DQB1*06:02, y not DQA1*02:01-DQB1*02, DQB1*03:01, DQB1*03:02, DQB1*06:02, or DQB1*06:03.

### Autoantibody Assays

Biochemically defined autoantibodies IAA, IA-2A, and GADA were analyzed with specific radio-binding method and ICAs with standard immunofluorescence method as previously described in (24–26). The cut-off levels used were 2.80 relative units (RU) for IAA, 5.36 RU for GADA and 0.78 RU for IA-2A determined as the level above 99 percentiles in more than 350 non-diabetic Finnish children. Islet cell antibodies were measured using an indirect immunofluorescence method using a cut-off value of 2.5 Juvenile Diabetes Foundation units.

### HLA Genotyping

Screening of HLA-risk alleles was performed as previously described (24–26). The initial HLA-DQB1 typing for risk-associated (DQB1*02, DQB1*03:02) and protective (DQB1*03:01, DQB1*06:02, and DQB1*06:03) alleles was complemented with DQA1 typing for DQA1*02:01 and DQA1*05 alleles in those with DQB1*02 without protective alleles or the major risk allele DQB1*03:02.

### DNA Extraction

DNA was extracted from fecal samples by using QIAamp Fast DNA Stool Mini Kit (Qiagen, Germany). In short, fecal samples (180–220 mg) were thawed in 1 ml of InhibitEX Buffer, vortexed for 1 min and incubated at 95°C for 10 min to enhance the lysis of hard-to-lyse taxa. After centrifugation, 200 μl of the supernatant was transferred to a new tube with proteinase K and Buffer AL and vortexed thoroughly. The lysate was incubated at 70°C for 10 min followed by addition of 0.3 vol. of absolute ethanol. Then, samples were vortexed, pipetted to the QIAamp spin column and centrifuged at 20,000 × g for 1 min. The column was washed with AW1 and AW2 Buffers, and the pure DNA was eluted in 200 μl of Buffer ATE and stored at −20°C. The quantity and quality of DNA was determined by using NanoDrop ND-1000 spectrophotometer (Thermo Fisher Scientific, Wilmington, DE, USA).

### Amplification of Bacterial 16s rRNA and Fungal ITS2 Region

The bacterial hypervariable regions V4-V5 of 16S rRNA gene were amplified using primers F519 (5′-CAGCMGCCGCGGTAATWC-3′) and R926 (5′-CCGTCAATTCCTTTRAGTTT-3′). The F519 primer contained an Ion Torrent pyrosequencing adapter sequence A (Thermo Fisher Scientific, USA), 9-bp unique barcode sequence and one nucleotide linker. The R926 primer contained an Ion Torrent adapter trP1 sequence. For fungal analysis, the ITS2 region was amplified using fITS7 (5′-GTGARTCATCGAATCTTTG-3′) and ITS4 (5′-TCCTCCGCTTATTGATATGC-3′) primers including the Ion Torrent pyrosequencing adaptor with a 10-bp barcode sequence to the ITS4 primer (27). PCR reactions were performed in three replicates, each containing 1x Phusion GC buffer, 0.4 μM of forward and reverse primers, 200 μM dNTPs, 0.5 U of Phusion High-Fidelity DNA Polymerase (Thermo Fisher Scientific) and 50 ng of genomic community DNA as the template and molecular grade water in a total reaction volume of 50 μl. For the bacteria, PCR cycling conditions were as follows: initial denaturation at 98°C for 3 min, 35 amplification cycles of 98°C for 10 s, 64°C for 10 s, and 72°C for 20 s, followed by a final extension step of 72°C for 7 min. For the fungi, the annealing temperature was adjusted to 56°C, while other PCR cycling conditions were kept unchanged. After the amplification, PCR products from the pooled triplicate reactions were purified with Agencourt AMPure XP beads (Agencourt Bioscience, MA, USA) and quantified with Agilent 2,100 Bioanalyzer (Agilent Technologies, CA, USA). The amplicons from each sample were then combined in equimolar concentrations to generate sequencing libraries. Sequencing was performed at Biocenter Oulu Sequencing Center with Ion Torrent PGM System on 316v2 chip using 400 bp chemistry (Thermo Fisher Scientific, USA).

### Bioinformatics Analysis

The bacterial and fungal sequencing data were processed using QIIME v.1.9.1 (28). The read data were quality controlled using the usearch quality filter pipeline, thus potential chimeric sequences were identified and removed with uchime (29). After filtering out low-quality and chimeric reads, the bacterial dataset consisted of 1.202 million reads across the 52 samples, with a mean of 23,120 reads per sample. The respective final fungal dataset comprised of 130,000 high-quality, chimera-free reads from the 52 samples, with a mean of 2,501 reads per sample. The sequences were clustered into the operational taxonomic units (OTUs) by a similarity threshold of 97% with usearch (30). Low-abundance OTUs (represented with <5 reads) were removed across the datasets. The taxonomy was assigned using the Greengenes 16S rRNA gene reference database (31) for bacteria (v.13_8) and UNITE ITS database for fungi (2019 release, v.8) (32). Prior to downstream analysis, the bacterial and fungal OTU tables were rarefied to 5,800 and 327 reads/sample, respectively, to avoid biases caused by variation in sequencing depth among samples (33). All the raw sequencing data were deposited in the NCBI-SRA database with an accession number SUB3267498.

We estimated beta diversity using the unweighted and weighted UniFrac distances (as well as non-phylogenetic Bray Curtis dissimilarity) between samples. Both UniFrac metrics incorporate phylogenetic distances between taxa, yet while the unweighted UniFrac compare microbial communities based on the presence/absence information, the weighted UniFrac also consider the differences in taxon abundance (34). Differences in the fungal and bacterial gut microbiota structure among children were visualized by Principal Coordinate analysis (PCoA) using EMPeror (35).

### Measurements of Fecal HBD2, Total IgA, and Calprotectin

Thawed fecal samples were mixed with extraction buffer and vortexed thoroughly. Then, the supernatant was collected and stored at −20°C until the analysis of total IgA, HBD2, and calprotectin levels. Total IgA concentrations were analyzed as previously described (36). HBD2 analyses, were performed with a commercial ELISA Kit according to the manufacturer's instructions (Immunodiagnostik AG, Bensheim, Germany). Fecal calprotectin levels were determined using Calprolab calprotectin ELISA test according to the manufacturer's instructions (Calpro AS, Lysaker, Norway).

### ELISA Analysis of Serum ASCA IgA/IgG Levels

Serum ASCA IgA and IgG concentrations were analyzed with a commercial ELISA kit according to the manufacturer's instructions (Demeditec, Germany), with the exception that 1:10 dilution of the serum samples were used. The samples below the lower limit of detection (LOD) were given an arbitrary value of 50% of the LOD being 0.5 U/ml for both ASCA IgA and IgG.

### Serum Cytokine Analysis

The serum concentrations of IFNG, IL-17A and IL-22 were analyzed using the Milliplex MAP Kit (HTH17MAG-14K) according to the manufacturer's recommendations (Merck-Millipore Corp., Billerica, MA, USA). Quantification of the markers was performed with a Bio-plex 200 Luminex-instrument and Bio-Plex Manager software (Bio-Rad, Sweden). The samples below Minimum detectable concentration (MinDC) DC were given an arbitrary value of 50% of MinDC.

### Statistical Analyses

The Graph Pad Prism 6.04 (Graph Pad Inc., La Jolla, California, USA), SPSS 22 (SPSS, Chicago, Illinois, USA) and JMP 13.0.0 statistical softwares were used for the statistical analyses, unless otherwise noted. Non-parametric Mann–Whitney *U*-test was used for comparisons between two groups. Groupwise comparisons were performed with the Kruskal–Wallis test. The correlations between the variables were analyzed with the non-parametric Spearman correlation test. Fisher's' exact test was used to analyze the distribution of autoantibody-positive children and disease progressors in different clusters. All statistical analyses were performed two-tailed. *P* < 0.05 was considered statistically significant. Despite of the matching of the autoantibody positive and negative children for age, T1D risk genotype and sex, the pairs were considered independent in the statistical analyses.

For the hierarchical clustering analysis, the relative abundance data was imported into JMP 13.0.0 (SAS Institute Inc. Cary, North Carolina, USA). All abundance values were treated as numerical values and Ward's hierarchical clustering was performed using standardized data with default settings. Statistical significance of samples grouping for beta diversity analysis was determined using the permutational multivariate analysis of variance (PERMANOVA) and the analysis of similarities (ANOSIM) (999 permutations) implemented by the *adonis* and *anosim* functions in the vegan R package (37).

## Results

### Study Design and Clinical T1D During the Follow-Up

In this study, we analyzed the gut microbiome of autoantibody positive and negative children with HLA conferred risk of T1D (Figure 1). We combined sequencing of the (1) bacterial 16S ribosomal RNA (rRNA) gene (2) and fungal internal transcribed spacer 2 (ITS2) region and coupled this with the (3) analysis of the markers of intestinal inflammation, namely fecal HBD2, secretory total IgA, and calprotectin. Blood samples were screened for the levels of ASCA IgA/IgG and circulating cytokines IFNG IL-17 and IL-22. Finally, the children in a cohort were followed for the development of clinical T1D (median of 8 years and 8 months). During the follow-up time, nine autoantibody positive children were diagnosed with T1D, and none of the autoantibody negative children developed T1D or autoantibodies.

### Fungal and Bacterial Dysbiosis in Children With Beta-Cell Autoimmunity

**Figure 2:**
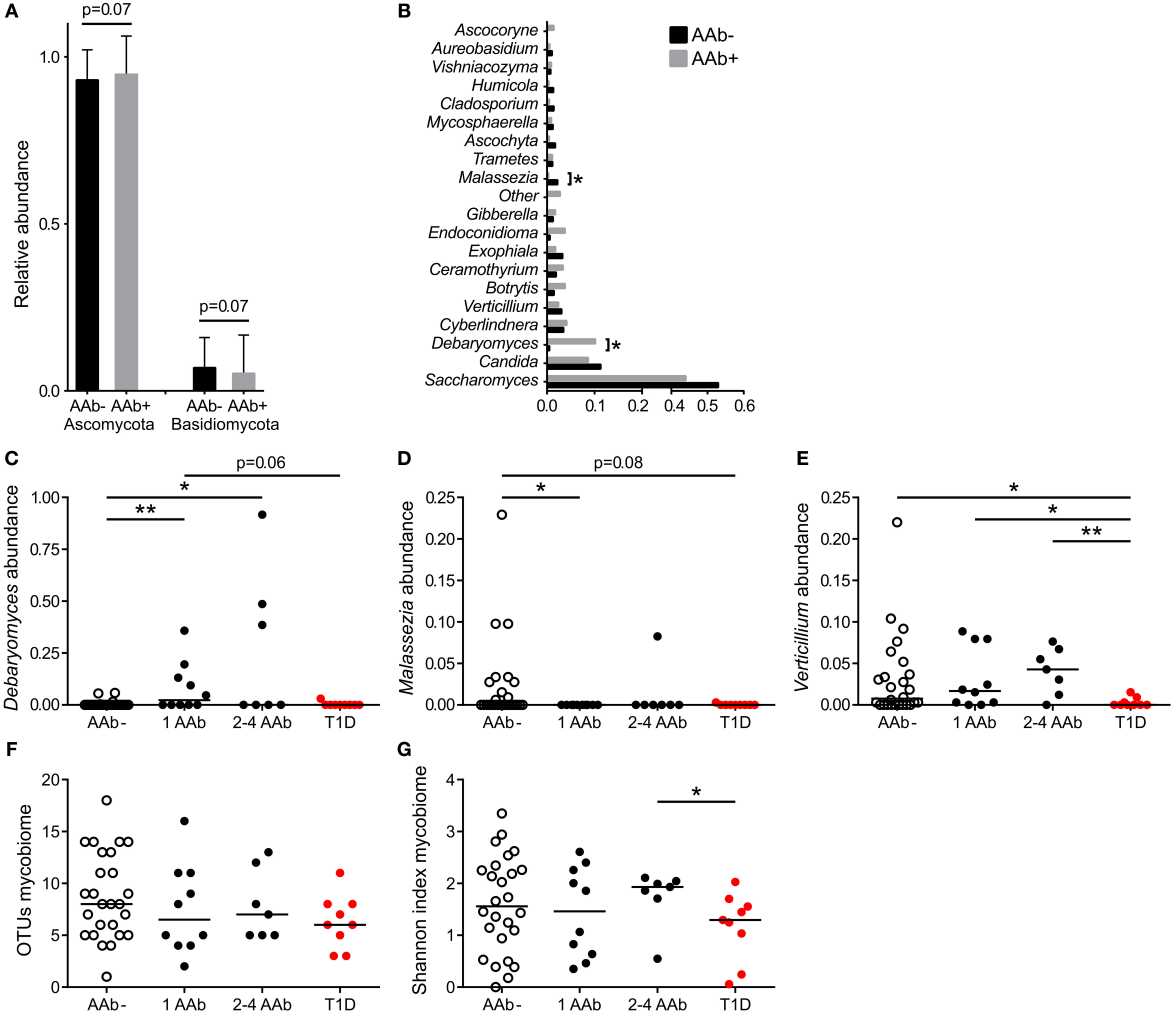
Fungal community characteristics in children with or without T1D-associated autoantibodies. **(A)** Relative abundance of fungal phyla Ascomycota (left columns) and Basidiomycota (right columns). Children without autoantibodies: black columns and children with autoantibodies: gray columns. **(B)** Relative abundance of 20 most abundant fungal genera in children with (gray bars) and without autoantibodies (black bars). **(C–E)** The relative abundances of *Debaryomyces* **(C)**, *Malessezia* **(D)**, and *Verticillium* **(E)** in healthy children without autoantibodies, in children with a different number of autoantibodies and in children who have progressed from autoantibody-positive state to clinical T1D. **(F,G)** Number of OTUs and Shannon diversity index in healthy children without autoantibodies, in children with a different number of autoantibodies and in children who have progressed from autoantibody-positive state to clinical T1D. Healthy children without autoantibodies are marked with open circles, children with 1–4 autoantibodies with black circles and children who have progressed to clinical disease with red circle. *p*-values were calculated with the Mann–Whitney *U*-test. *p < 0.05, **p < 0.01.

The gut mycobiota was composed of two fungal phyla, Ascomycota and Basidiomycota, but was dominated by Ascomycota (average 93%) at the phylum level and by *Saccharomyces* at the genus level (average 43%) (Figures 2A,B). All 52 study subjects were positive for Ascomycota, and 29 of them (56%) positive for Basidiomycota (11 of 26 autoantibody positive and 18 of 26 negative individuals, 42 and 69%, respectively). Children with autoantibodies had marginally increased abundance of Ascomycota and decreased levels of Basidiomycota (Figure 2A). The most frequently observed genera were *Saccharomyces* (found in all 52 individuals), *Candida* (found in 9 of 26 autoantibody positive and 13 of 26 autoantibody negative individuals, 35 and 50%) and *Debaryomyces* (found in 9 of 26 autoantibody positive and 4 of 26 autoantibody negative individuals, 35 and 15%). Children with autoantibodies had increased abundance of *Debaryomyces* and decreased abundance of *Malassezia* (Figures 2B–D). The number of children positive for *Debaryomyce*s or *Malassezia* was, however, low, and 25.0 and 23.1% of the studied children were positive for *Debaryomyces (13/52)* and *Malassezia (12/52)*, respectively. The autoantibody-positive children who developed clinical T1D during the follow-up had significantly decreased abundance of genus *Verticillium* compared to children with or without autoantibodies (Figure 2E). *Verticillium* positivity was found in 16 of 26 (62%) autoantibody positive and 17 of 26 (65%) autoantibody negative children. We did not observe differences in the number of OTUs between children with or without autoantibodies (Figure 2F). Children who developed T1D had decreased fungal diversity (Shannon) compared to children with multiple autoantibodies (Figure 2G). Principal coordinate analysis based on weighted and unweighted UniFrac distances did not show clear differences between autoantibody-negative and autoantibody-negative children (Supplementary Figures 1C,D). The fungal community composition is consistent with the gut mycobiome communities previously reported for humans (18, 38, 39). List of the most abundant fungal species shared among the autoantibody-negative and positive children is presented in Supplementary Table 1.

**Figure 3:**
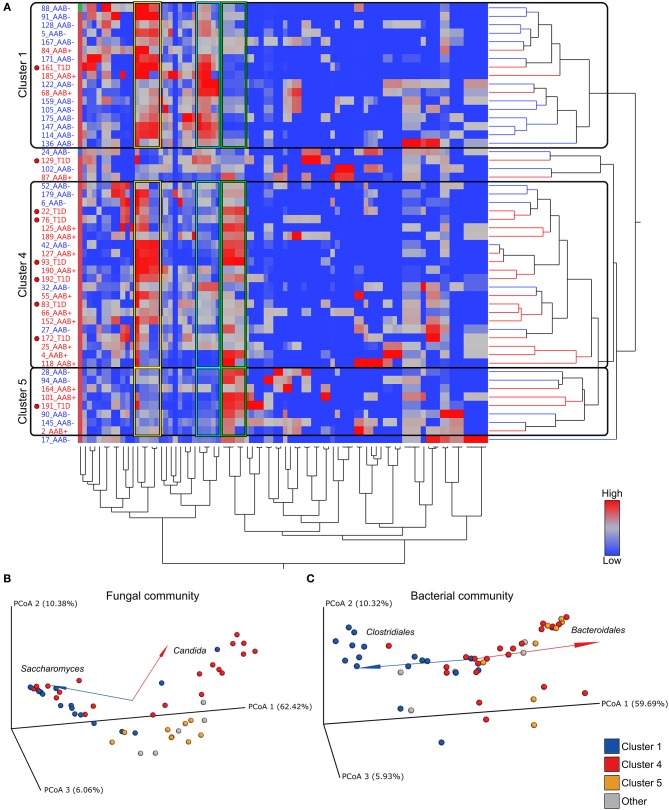
Hierarchical clustering over bacterial and fungal taxa and PCoA plots of fungal and bacterial communities. **(A)** Heatmap showing the clustering of the relative abundances. The clustering resulted in three major clusters (Clusters 1, 4, and 5) defined by differential abundance of fungi belonging to the phylum Ascomycota and the bacterial phyla Firmicutes and Bacteroidetes. Clusters 1, 4, and 5 are encircled with black borders. In the heatmap children with autoantibodies are denoted with AAb+ (red font) and autoantibody-negative children with AAb (blue font). Children who have progressed from autoantibody-positive state to clinical disease are denoted with T1D and a red circle in the heatmap. Color intensity of the heatmap increases with the taxa relative abundance from low (blue) to high (red). **(B,C)** Principal coordinate analysis (PCoA) biplots (incorporate taxonomy information) on weighted UniFrac distances between the fungal **(B)** and bacterial **(C)** gut microbial communities profiles of children at risk of the type 1 diabetes development. Each point represent a single sample and is colored according to the major taxa clusters (Clusters 1, 4, 5, and other), as defined by the hierarchical clustering analysis. Order and genus-level taxonomy displayed by biplot arrows illustrates that the abundance of **(B)** the *Saccharomyces* and *Candida* contribute to the separation of Cluster 1 and Cluster 4, and **(C)** Clostridiales and Bacteroidales contribute to the distinct clustering patterns of Cluster 1 in comparison to Clusters 4 and 5. *p*-values were calculated with the Mann–Whitney *U*-test. *p < 0.05, **p < 0.01. Grouping significance was determined using the PERMANOVA (999 permutations) with the *adonis* function in the vegan R package (*p* < 0.001).

Next, we addressed the whole microbiota and performed a combinational analysis of fungal and bacterial communities. Our hierarchical clustering analysis based on the combined fungal and bacterial data revealed the presence of three major clusters as defined by different combinations of fungi, belonging to the phylum Ascomycota, and the bacterial phyla Bacteroidetes and Firmicutes (Figures 3A–C). The fungal and bacterial community structures differed among children assigned to distinct clusters (*p* < 0.001, PERMANOVA) (see the Supplementary Table 2). Cluster 1 (*n* = 17, 33%) was characterized by high abundance of Clostridiales and low abundance of Bacteroidales in combination with high abundance of *Saccharomyces* (Figures 3B,C and Supplementary Figure 2A). Conversely, Clusters 4 (*n* = 22, 42%) and 5 (*n* = 8, 15%) were characterized by high abundance of Bacteroidales and low abundance of Clostridiales, and Cluster 4 also showed high abundance of *Candida* compared to Cluster 1 (Figures 3B,C and Supplementary Figure 2B). Although, abundance of Ascomycota was high in Clusters 1 and 4, the abundance of *Saccharomyces* and *Candida* differed significantly between the clusters (Figure 3B, Supplementary Figures 2A,B and Supplementary Tables 2, 3). Relative abundances of *Debaryomyces, Malassezia* and *Verticillium* in Clusters 1, 4 and 5 are shown in Supplementary Figures 2C–E. The relative abundances of *Saccharomyces* and *Candida* in children with or without autoantibodies are shown in Supplementary Figures 2F,G. Analysis of similarities of fungal and bacterial communities between different clusters, revealed that Clusters 4 and 5 differed from Cluster 1 for both fungal and bacterial communities (Supplementary Table 3). Host gender had no statistically significant (*p* > 0.05, PERMANOVA) explanatory effect on the fungal and bacterial communities (Supplementary Table 2). Children age and HLA-risk class made a statistically significant contribution to the total variation in the bacterial community (*p* = 0.02, *R*^2^ = 0.08, *p* < 0.001, *R*^2^ = 0.14, for the host age and HLA-risk class, respectively), albeit with a relatively low coefficient of determination, and non-significant (*p* > 0.05) contribution of both factors to the mycobiome community structure (Supplementary Table 2).

Next, we analyzed the distribution of autoantibody positive and negative children in these three major microbiome clusters. The children with beta-cell autoimmunity were enriched in Clusters 4 and 5 (Cluster 1 vs. Cluster 4, *p* = 0.004, Cluster 1 vs. Cluster 5, *p* = ns and Cluster 4 vs. Cluster 5, *p* = ns, Fisher's exact test), while the children negative for beta-cell autoimmunity, and thus considered healthy children, were enriched in Cluster 1 (shown in Figure 3A and in Table 2). By the end of the follow-up, 6 out of 22 children in Cluster 4 (27%) had developed T1D, one of eight children in Cluster 5 (13%), and just one of 17 children in Cluster 1 (6%) were diagnosed for T1D (Cluster 4 vs. 5, *p* = 0.64 and Cluster 4 vs. 1, *p* = 0.11, respectively). One child who was not assigned in any of the three major clusters developed T1D.

**Table 2:** Distribution of autoantibody-negative children, children with one or multiple autoantibodies and disease progressors in the different Clusters.

	**Cluster 1**	**Cluster 4**	**Cluster 5**
AAb-	13 (76.5%)	6 (27.2%)	4 (50%)
1 AAb	1 (5.8%)	7 (31.8%)	2 (25%)
2-4 AAbs	2 (11.7%)	3 (13.6%)	1 (12.5%)
T1D progressors	1 (5.8%)	6 (27.2%)	1 (12.5)
Total	17	22	8

*Relative proportions of different study groups within the Clusters are presented in the parentheses*.

### Intestinal Inflammation in Children With Fungal and Bacterial Dysbiosis

**Figure 4:**
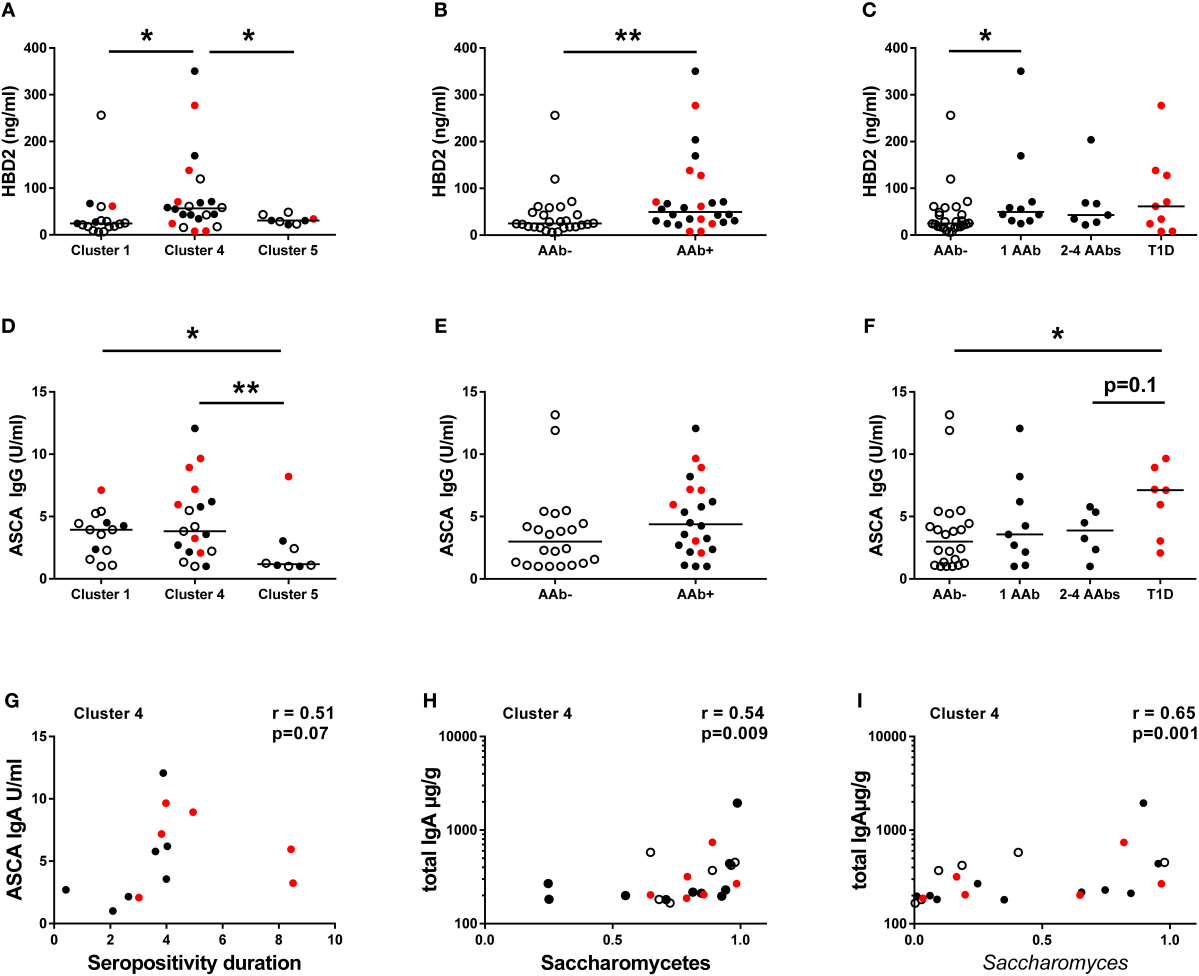
Inflammatory markers in different clusters and in children with or without beta-cell autoimmunity. **(A)** Fecal concentrations of HBD2 in the Clusters 1, 4, and 5. HBD2 levels were higher in the species Cluster 4 compared to Clusters 1 and 5. **(B)** Children with autoantibodies had elevated levels of fecal HBD2 compared to autoantibody-negative children. **(C)** Children with only one autoantibody had elevated levels of fecal HBD2 compared to autoantibody-negative children. **(D)** Serum ASCA IgG concentrations were significantly higher in the Clusters 1 and 4 compared to the Cluster 5. **(E)** Serum ASCA IgG concentrations in AAb- and in AAb+ children. **(F)** The autoantibody-positive children who have progressed to clinical disease had elevated serum ASCA IgG antibodies compared to autoantibody-negative children. **(G)** The relationship between serum ASCA IgA levels and seropositivity duration in the species Cluster 4. **(H)** The relationship between fecal total IgA and relative abundance of Saccharomycetes in the Cluster 4. **(I)** The relationship between fecal total IgA and relative abundance of *Saccharomyces* in the Cluster 4. Healthy children without autoantibodies are marked with open circles, children with 1–4 autoantibodies with black circles and children who have progressed to clinical disease with red circle. Horizontal lines represent median values. *p*-values were calculated with the Mann–Whitney *U*-test. Correlations were calculated with the Spearman rank correlation test.*p < 0.05, **p < 0.01.

To address the relation between the composition of the intestinal microbiota and host inflammatory response, we analyzed fecal concentrations of HBD2, which is an antimicrobial peptide secreted by the epithelial cells in response to microbial stimulus and IL-17/IL-22 pathway activation (40). Interestingly, the children in Cluster 4 had higher fecal HBD2 levels compared to children in Clusters 1 and 5 (Figure 4A), and consequently autoantibody positive children had higher levels of fecal HBD2 than autoantibody-negative children (Figure 4B). However, the autoantibody-positive children in Cluster 4 had higher levels of fecal HBD2 than those in Cluster 5 (Supplementary Figure 3). Notably, the children with only one autoantibody showed higher levels of fecal HBD2 compared to autoantibody-negative children (Figure 4C). Fecal calprotectin levels did not differ between the autoantibody-negative or positive children or between the species clusters (Supplementary Figures 4D–F).

Since the high abundance of order Saccharomycetales (the most abundant genera in the data set belonging to the Saccharomycetales were: *Saccharomyces, Candida*, and *Debaryomyces*) was a key feature of the children in Cluster 1 and 4, we measured the levels of serum ASCA shown earlier to be associated with fungal dysbiosis in Crohn's disease (41, 42). ASCA IgG levels were significantly higher in children belonging to Clusters 1 and 4 compared to the children in Cluster 5 (Figure 4D), suggesting that the ASCA IgG production is indeed induced by high abundance of Saccharomycetes, which was observed in children in the Clusters 1 and 4. ASCA IgG levels did not, however, correlate with the abundance of Saccharomycetes or *Saccharomyces* in either the children in Cluster 1 or 4 (*p* = 0.33 and *p* = 0.73). Indeed, the highest ASCA IgG levels were observed in the children who progressed to clinical T1D, irrespective of the assigned Cluster (Figures 4E,F). Moreover, ASCA IgG levels showed a tendency of positive correlation with the duration of autoantibody positivity in the Cluster 4 children (Figure 4G). ASCA IgA levels did not differ significantly between the clusters, but it should be noted that the majority of individuals studied had ASCA IgA levels below the lower limit of detection (Supplementary Figures 4G–I). We also observed a positive correlation between fecal total IgA levels and Saccharomycetes (and also *Saccharomyces*) abundance in Cluster 4, suggesting a local intestinal immunostimulatory effect of *Saccharomyces* in the children in Cluster 4 (Figures 4H,I).

### Bacteroidetes vs. Firmicutes Ratio as a Regulator of Systemic Low-Grade Inflammation

**Figure 5:**
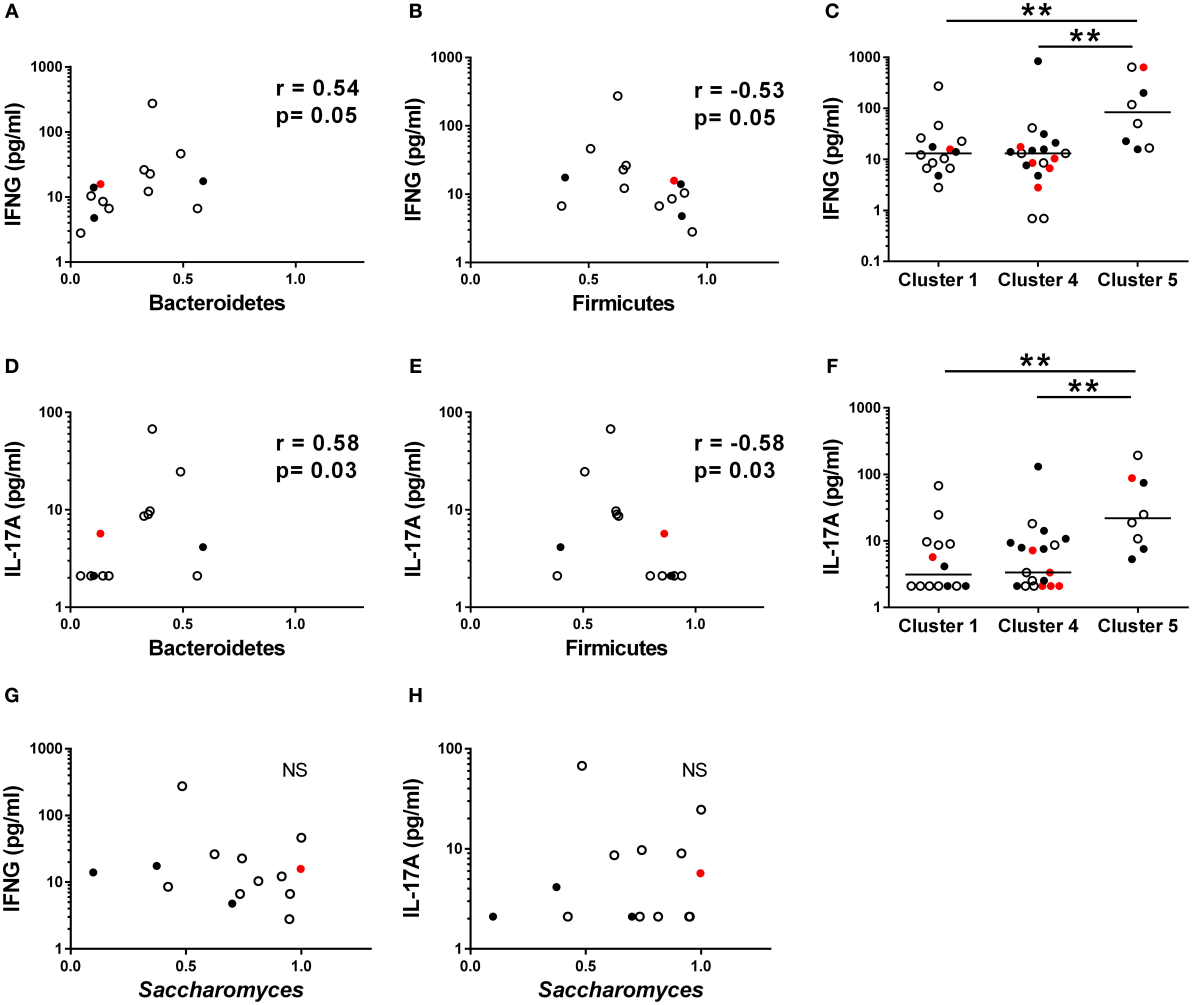
Serum cytokine concentration in different clusters. **(A,B)** The relationships between serum IFNG and relative abundance of fecal Bacteroidetes and Firmicutes in the Cluster 1. **(D,E)** The relationships between serum IL-17A and relative abundance of fecal Bacteroidetes and Firmicutes in the Cluster 1. **(C,F)** IFNG and IL-17A levels were significantly higher in the Cluster 5 compared to the Clusters 1 and 4. **(G,H)** The relationships between serum IFNG and relative abundance of fecal Bacteroidetes and Firmicutes in the Cluster 1. Horizontal lines represent median values. *p*-values were calculated with the Mann–Whitney *U*-test. Correlations were calculated with the Spearman rank correlation test.*p < 0.05, **p < 0.01.

Given that Th1 and Th17 immunity have been earlier associated with fungal dysbiosis (43) and T1D (6, 9, 44), we measured concentrations of the circulating cytokines IFNG, IL-17A, and IL-22, in the serum samples of the study participants. In Cluster 1, enriched with the autoantibody negative children and representing thus healthy children, both IFNG and IL-17A concentrations correlated positively with the abundance of Bacteroidetes (Figures 5A,D) and inversely with the abundance of Firmicutes (Figures 5B,E). In agreement with this, we found that the children in Cluster 5, characterized by increased Bacteroidetes to Firmicutes ratio, showed increased levels of circulating IFNG and IL-17A (Figures 5C,F). In Cluster 4, enriched with the autoantibody positive children with intestinal inflammation, no correlations were observed between circulating cytokines and microbiota composition. No correlation was found between the relative abundance of *Saccharomyces* and circulating cytokines (Figures 5G,H) in Cluster 1. Serum IFNG and IL-17A concentrations in children with or without beta-cell autoimmunity are shown in Supplementary Figure 5.

## Discussion

In a prospective study including 52 children at risk of T1D, we show that intestinal dysbiosis is associated with the later development of T1D, and is characterized by altered fungal and bacterial communities and intestinal inflammation. Signs of intestinal inflammation and increased permeability have been earlier associated with clinical T1D (15, 26, 45–47). Bosi et al. (48) have shown increased intestinal permeability also in pre-diabetes (48). To our knowledge, however, these kinds of associations between the composition of the intestinal microbiome, intestinal inflammatory markers, and their potential contribution to the disease progression have not been reported earlier in children at risk of T1D.

The combined hierarchical clustering analysis of fungal and bacterial taxa provided the separation of the autoantibody positive children into two groups which differed in the progression to T1D during the follow-up. Long-term follow-up studies are rare, but show that nearly all children positive for multiple autoantibodies and genetic risk of T1D develop the clinical disease in 15–20 years (49). Thus, the children who developed T1D in our cohort can be considered as rapid progressors (Cluster 4) in comparison to the autoantibody positive children who remained healthy (Cluster 5). The levels of fecal HBD2 indicating epithelial intestinal inflammation were the highest in the children with rapid disease progression (i.e., Cluster 4) suggesting that intestinal inflammation is a marker of disease progression. The altered bacterial community, which was seen in Clusters 4 and 5, is likely associated with the development of beta-cell autoimmunity as such. We did not find significant differences in fecal calprotectin levels between the clusters, or children with or without beta-cell autoimmunity, suggesting that neutrophil activation is not mediating intestinal inflammation. The levels of fecal calprotectin in our study cohort were comparable to the levels reported earlier in healthy children (50).

Our mycobiome data suggest that fungal dysbiosis could play a role in the disruption of intestinal homeostasis and development of subclinical low-grade intestinal inflammation, which associate with the disease progression.

Altered abundances of fungi assigned to *Malassezia* and *Debaryomyces* taxa were found in children with beta-cell autoimmunity, and a decreased abundance of fungi assigning to *Verticillium* genus was observed in children who later progressed to clinical T1D. Fecal *Debaryomyces* and *Malassezia* have been occasionally reported in human studies, but currently there is no consensus whether these fungal taxa are permanent residents of human intestinal microbiota (17). *Verticillium* has been reported very rarely in humans (51, 52). In line with a recent comprehensive review on human gut mycobiota (18), *Saccharomyces*, and *Candida* genera were the most frequently observed fungal taxa with the highest relative abundances among the children in our cohort.

Importantly, the high relative abundance of *Candida* was characteristic to the fungal dysbiosis that separated the autoantibody positive children with rapid disease progression from the rest of the autoantibody positive children who did not develop T1D, and from the autoantibody negative children. Thus, the increased colonization with *Candida* could be an important factor contributing to the intestinal inflammation and further progression to T1D.

*Candida* is a member of the healthy intestinal microbiome, and the degree of *Candida* colonization is regulated by host related factors, such as epithelial integrity and IL-17/IL-22 immunity, and by the composition of the commensal bacterial community (43, 53–56). Commensal bacteria interfere with fungal colonization and compete for surface and nutrients, and bacteria-produced short chain fatty acids (SCFAs) can inhibit *Candida* virulence by preventing yeast-hyphal transition (57). Bacteria can also modulate epithelial barrier function and integrity by their SCFA metabolites, such as butyrate, and by regulation of the production of mucus, IL-22, and anti-microbial peptides (54, 55, 58). Thus, the low relative abundance of Clostridiales and butyrate-producing bacteria found in the autoantibody positive children could contribute to the increasing colonization by *Candida*. Low abundance of butyrate-producing bacteria has been reported also in autoantibody positive children in earlier studies (10, 59). However, despite of the low abundance of Clostridiales in both Clusters 4 and 5, increase in *Candida* was only seen in Cluster 4 including the rapid progressors. In humans, efficient control and eradication of *Candida* requires the activation of IL17A and IFNG-producing Th17 cells (60, 61). It is thus possible that the observed high levels of circulating IL-17 and IFNG in Cluster 5 could provide resistance to fungal colonization. Indeed, the abundance of *Saccharomyces* was significantly decreased in Cluster 5 with the highest levels of circulating IL-17 and IFNG. The increase in IL-17 and IFNG in Cluster 5 can actually be a consequence of the bacterial dysbiosis characterized by low abundance of Firmicutes and high abundance of Bacteroidetes, similarly as seen in the autoantibody negative children in Cluster 1, who showed a positive correlation with circulating IL-17 and IFNG and a high Bacteroidetes to Firmicutes ratio. Instead, in Cluster 4, the children did not respond to bacterial dysbiosis with IL-17 and IFNG upregulation, which could provide a niche for *Candida* colonization, and finally to the local mucosal inflammation in the intestine. When we analyzed the relationship between relative abundances of *Saccharomyces* or *Candida* and circulating cytokines, we did not observe significant correlations in children in Cluster 1 underlining the importance of Bacteroidetes and Firmicutes in regulation of IFNG and IL-17 responses in a healthy state.

The human intestinal microbiota is a dynamic system of bacteria, fungi, protists and viruses that co-exist and thus, may converge in response to various external or internal stimuli. Interkingdom associations between bacteria and fungi within gut microbiome have been previously reported in Crohn's disease, where different fungal genera were positively correlated with several bacterial taxa (21, 22). The potency of the mycobiota to regulate the bacterial compartment is suggested by animal studies showing that restoration of the bacterial compartment after antibiotic depletion of bacteria was strongly influenced by colonization with *C. albicans* (62). In a mouse model of liver injury, administration of *Saccharomyces boulardii* changed the composition of intestinal bacterial compartment by increasing the relative abundance of Bacteroidetes and decreasing the relative abundance of the bacteria belonging to the Firmicutes (63). Interestingly, Enterobacteriaceae, such as *Escherichia coli* has been shown to cooperate with yeast to favor their colonization and inflammatory properties in the intestine in an animal model of ulcerative colitis (64). The implication of these data is that intestinal fungal and bacterial communities can regulate each other, but the understanding of the ecological network and its cross-talk with the host remain largely unknown.

We recognize that our study has limitations, such as the relatively low number of studied individuals and the lack of longitudinally collected fecal and blood samples during the follow-up. Although we observed the alterations in the bacterial and fungal communities and in the markers of intestinal inflammation in the samples collected already years before the signs of clinical T1D, longitudinal sampling and mechanistic studies would have strengthened the study, which is currently descriptive in nature. Temporal relationship of bacterial and fungal dysbiosis linked to the development of intestinal inflammation, beta-cell autoimmunity and T1D needs further prospective and mechanistic studies, and as always, the results should be to be replicated in independent cohorts before the findings can be generalized.

There is an urgent need for new biomarkers that could be used for the identification of the individuals with increased risk of beta-cell autoimmunity and for the prediction of the progression from autoantibody positivity to T1D. It is tempting to speculate that an increased *Candida* abundance and associated intestinal inflammation, measured by increased levels of ASCA and HBD2 levels, could provide new tools for the more accurate prediction of T1D.

Longitudinal studies are needed to provide information on the sequential order of the changes in gut microbiota, intestinal inflammation and peripheral immunity leading to beta-cell autoimmunity and clinical T1D. Despite the limitations in the current study, our findings show that intestinal mycobiota is diverse and can be analyzed in pediatric fecal samples. Our results emphasize the importance of the fungal dysbiosis, in addition to bacterial dysbiosis, in shaping intestinal homeostasis and inflammation preceding T1D.

## Data Availability Statement

All microbiome data was uploaded to NCBI BioProject database with accession number PRJNA420169 & PRJNA420171. The other datasets are available on request to the first author (JH).

## Ethics Statement

The studies involving human participants were reviewed and approved by the Ethics Committee of the Hospital District of Helsinki and Uusimaa. Written informed consent to participate in this study was provided by the participants' legal guardian/next of kin.

## Author Contributions

JH, JK, and OV conceived the original idea. JH and OV wrote the manuscript. JK, AL, and MT were responsible for DNA analyses and bioinformatics of the microbiological studies. DM was responsible for clustering analyses. AV performed serum ASCA Ig and cytokine analyses. JH, LO, JK, AL, MT, AV, CF, and DM analyzed the data. TR and KL coordinated the study subject recruitment and sample collection. MK contributed to the study subject recruitment and edited the manuscript. JK, AL, and AP contributed to the writing and critically reviewed the manuscript. MK and TH were responsible for the autoantibody analyses. JI was responsible for HLA typing. LO performed the HBD2, total IgA, and calprotectin analyses. OV was responsible for the study design.

### Conflict of Interest

DM was employed by the company AstraZeneca, but AstraZeneca did not have a role in the study. The remaining authors declare that the research was conducted in the absence of any commercial or financial relationships that could be construed as a potential conflict of interest.

## Supplementary Material

Supplementary Figure 1 Diversity of the fungal communities. **(A)** Rarefaction curves showing the alpha diversity in the fungal community across all the samples, and **(B)** in children with 1-4 β-cell autoantibodies (red curve) and autoantibody negative samples (blue curve). Each curve is showing an average number of OTUs found in the given number of sampled sequences after rarefaction at the depth of 327 sequences per sample. Principal coordinate analysis (PCoA) plots based on the weighted **(C)** and unweighted **(D)** UniFrac distances between the fecal fungal communities in children with (red dots) or without (blue dots) autoantibodies.

Supplementary Figure 2 Relative abundances of fungal genera *Saccharomyce*s, *Candida, Debaryomyces, Malassezia* and *Verticillium* in different Clusters and in children with or without T1D-associated autoantibodies. Relative abundances of *Saccharomyces* **(A)** and *Candida* **(B)** in major Clusters 1, 4, and 5. **(C–E)** relative abundances of *Debaryomyces, Malassezia* and *Verticillium* in fecal samples from children in major Clusters. **(F,G)** Healthy children without autoantibodies are marked with open circles, children with 1–4 autoantibodies with black circles and children who have progressed to clinical disease with red circle. *P*-values were calculated with the Mann–Whitney *U*-test. **p* < 0.05, ^**^*p* < 0.01.

Supplementary Figure 3 Intestinal inflammation in children with autoantibodies in Clusters 4 and 5. AAb+ children in the Cluster 5 had significantly lower fecal HBD2 levels compared to AAb+ children in the Cluster 4. Horizontal lines represent median values. Children with 1–4 autoantibodies are marked with black circles and children who have progressed to clinical disease with red circle. Dotted line represents the highest observed value in the Cluster 5. 81% of the individuals in the cluster 4 had HBD2 level higher than the highest value in the Cluster 5. *p*-values were calculated with the Mann–Whitney *U*-test. **p* < 0.05.

Supplementary Figure 4 Inflammatory markers in different clusters and in children with or without beta-cell autoimmunity. **(A)** Fecal concentration of total IgA in the major species Clusters 1, 4, and 5. **(B)** Fecal total IgA concentrations in AAb+ children and AAb- children. **(C)** Fecal concentration of total IgA in AAb-children, in children with a different number of autoantibodies and in children who have progressed to clinical T1D. **(D)** Fecal concentration of calprotectin in the major species Clusters 1, 4, and 5. **(E)** Fecal calprotectin concentrations in AAb+ children and AAb- children. **(F)** Fecal concentration of calprotectin in AAb-children, in children with a different number of autoantibodies and in children who have progressed to clinical T1D. **(G)** Serum ASCA IgA antibody levels in the different species Clusters. **(H)** Serum ASCA IgA levels in AAb- and AAb+ children. **(I)** Serum ASCA IgA concentrations in AAb- children and in children with only one or multiple autoantibodies and in those children who have progressed to clinical disease. **(J)** Serum IL-22 levels in different species clusters. **(K)** IL-22 levels in AAb- and AAb+ children. **(L)** IL-22 levels in AAb- children and in children with different numbers of autoantibodies.

Supplementary Figure 5 **(A,B)** Serum IFNG and IL-17A concentration in children with or without beta-cell autoimmunity.

Supplementary Table 1 The top 25 most abundant fungal species (based on the observed sequences) shared among the autoantibody-negative children and children with autoantibodies.

Supplementary Table 2 Summary of the permutational multivariate analysis of variance (PERMANOVA). PERMANOVA statistical tests were performed on the weighted UniFrac distances and Bray Curtis dissimilarity between the (1) fungal and (2) bacterial gut microbial communities profiles of children at risk of the type 1 diabetes development. Statistical tests were run using the adonis function in R package vegan (with 999 permutations). The significant (<0.05) values shown in bold.

Supplementary Table 3 Summary of the analysis of similarities (ANOSIM). ANOSIM statistical tests were performed on the weighted UniFrac distances and Bray Curtis dissimilarity between the (1) fungal and (2) bacterial gut microbial communities profiles of children at risk of the type 1 diabetes development. Statistical tests were run using the anosim function in R package vegan (with 999 permutations). The Benjamini-Hochberg False Discovery Rate (FDR) correction for multiple testing was used to calculate q-values. The significant (<0.05) values shown in bold.
