# Supplementary figures and images for: Fungal Dysbiosis and Intestinal Inflammation in Children With Beta-Cell Autoimmunity
Source: Front Immunol. 2020 Mar 19;11:468. doi: 10.3389/fimmu.2020.00468 (PMC7103650; doi:10.3389/fimmu.2020.00468)

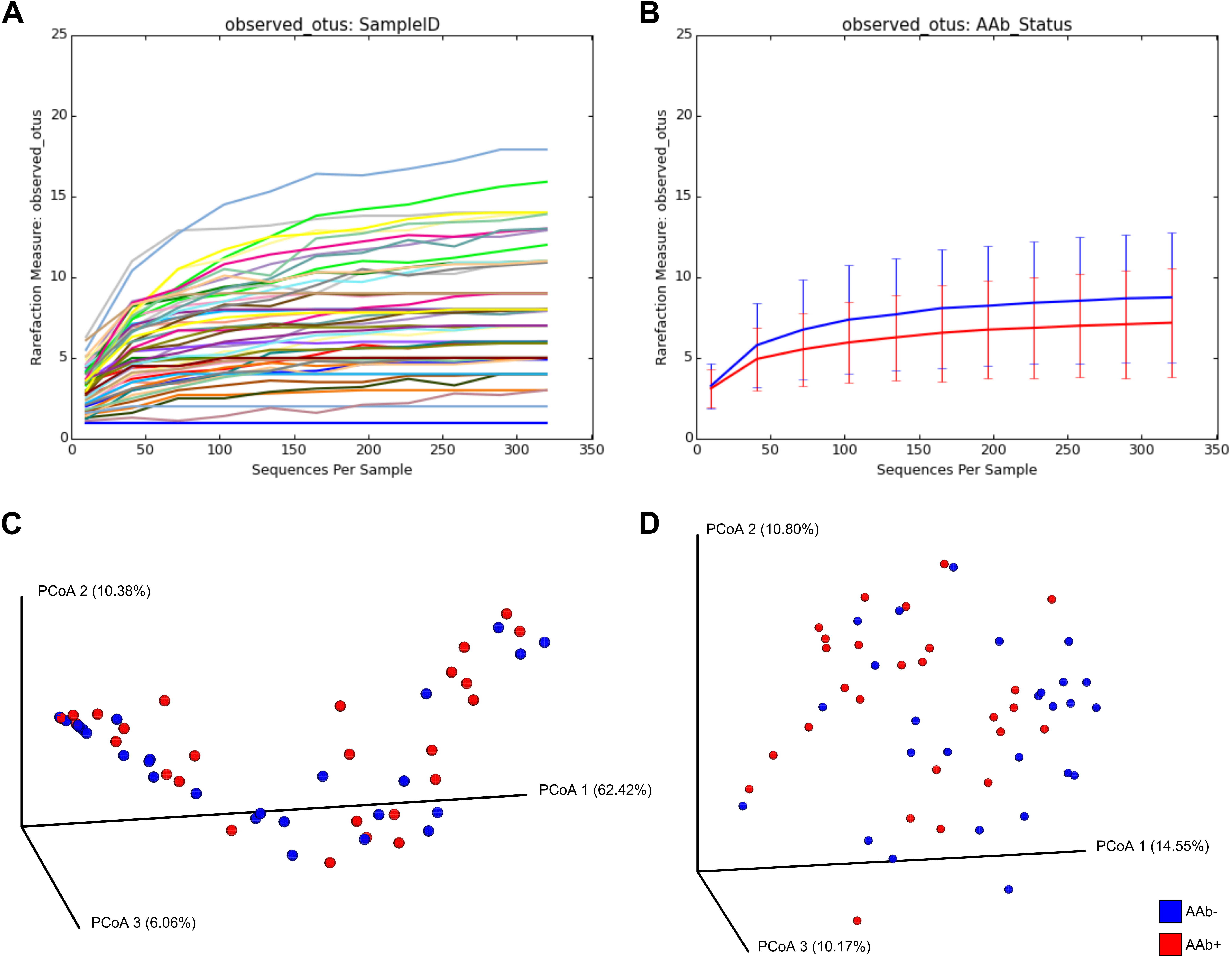

Supplement: Supplementary Figure 1 — Diversity of the fungal communities. (A) Rarefaction curves showing the alpha diversity in the fungal community across all the samples, and (B) in children with 1-4 β-cell autoantibodies (red curve) and autoantibody negative samples (blue curve). Each curve is showing an average number of OTUs found in the given number of sampled sequences after rarefaction at the depth of 327 sequences per sample. Principal coordinate analysis (PCoA) plots based on the weighted (C) and unweighted (D) UniFrac distances between the fecal fungal communities in children with (red dots) or without (blue dots) autoantibodies. [file Image_1.TIF]

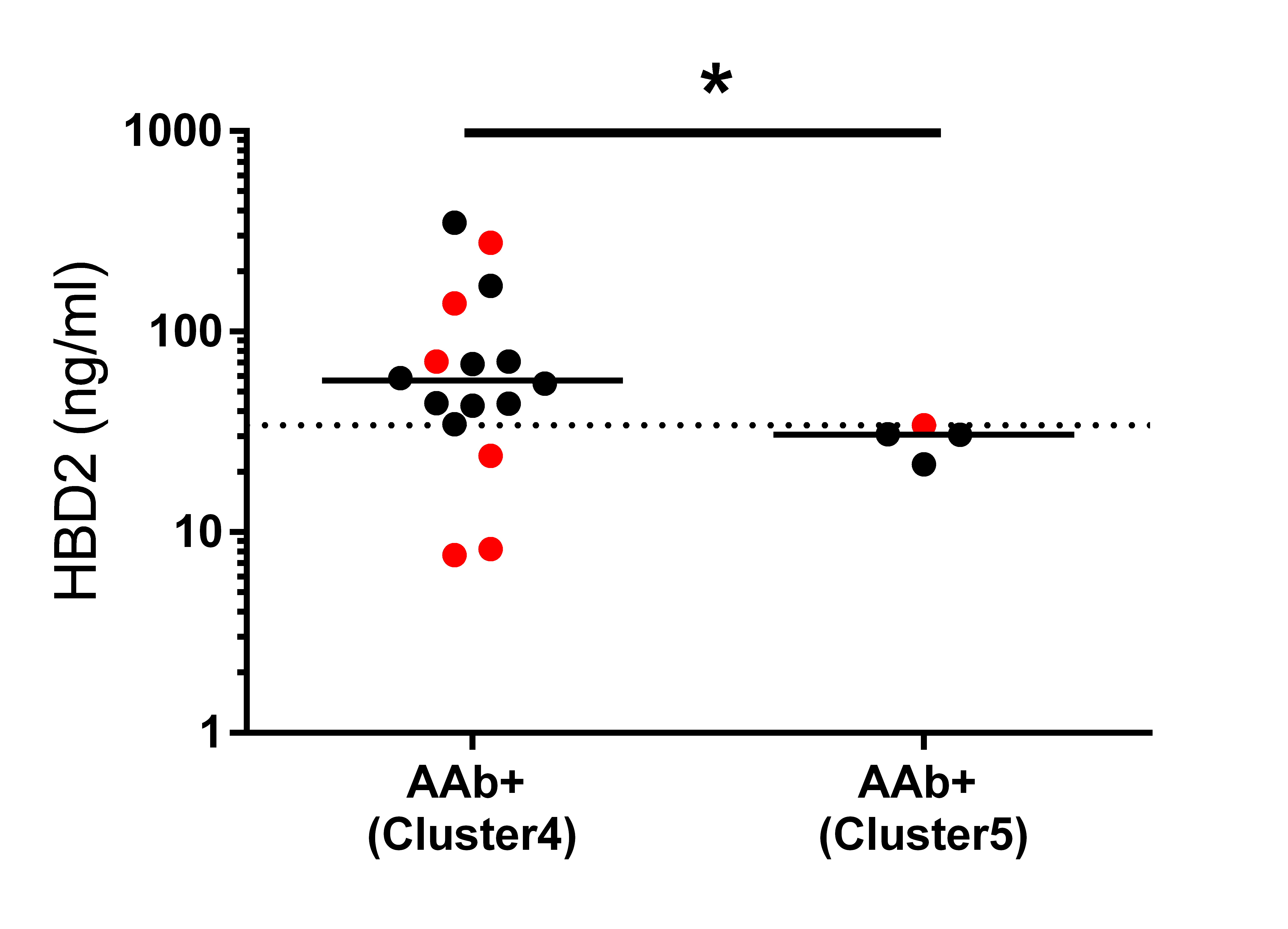

Supplement: Supplementary Figure 3 — Intestinal inflammation in children with autoantibodies in Clusters 4 and 5. AAb+ children in the Cluster 5 had significantly lower fecal HBD2 levels compared to AAb+ children in the Cluster 4. Horizontal lines represent median values. Children with 1–4 autoantibodies are marked with black circles and children who have progressed to clinical disease with red circle. Dotted line represents the highest observed value in the Cluster 5. 81% of the individuals in the cluster 4 had HBD2 level higher than the highest value in the Cluster 5. p-values were calculated with the Mann–Whitney U-test. *p < 0.05. [file Image_3.TIF]

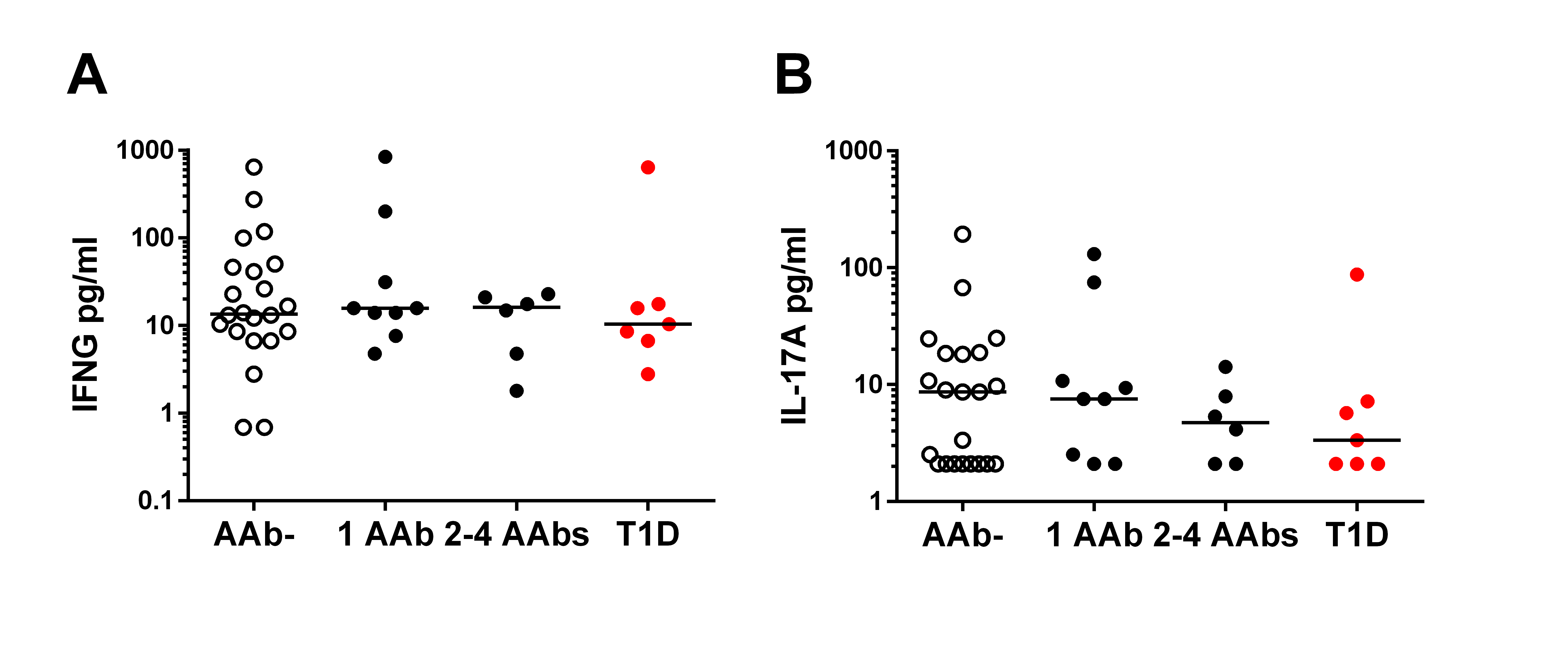

Supplement: Supplementary Figure 5 — (A,B) Serum IFNG and IL-17A concentration in children with or without beta-cell autoimmunity. [file Image_5.tif]
