# Supplementary material for: Fungal Dysbiosis and Intestinal Inflammation in Children With Beta-Cell Autoimmunity
Source: Front Immunol. 2020 Mar 19;11:468. doi: 10.3389/fimmu.2020.00468 (PMC7103650; doi:10.3389/fimmu.2020.00468)
Supplement: Supplementary Table 1 — The top 25 most abundant fungal species (based on the observed sequences) shared among the autoantibody-negative children and children with autoantibodies. [file Table_1.DOCX]

**Supplementary Table 1.** The top 25 most abundant fungal species (based on the observed sequences) shared among the autoantibody-negative children and children with autoantibodies.

| **Fungal species** | **AAb +** | **AAb -** | **Total** |
| --- | --- | --- | --- |
| *Saccharomyces cerevisiae* | 3737 | 4502 | 8239 |
| *Candida tropicalis* | 654 | 846 | 1500 |
| *Debaryomyces hansenii* | 865 | 43 | 908 |
| *Cyberlindnera jadinii* | 295 | 235 | 530 |
| *Verticillium leptobactrum* | 204 | 262 | 466 |
| *Ceramothyrium carniolicum* | 286 | 164 | 450 |
| *Botrytis caroliniana* | 324 | 126 | 450 |
| *Endoconidioma populi* | 321 | 54 | 375 |
| *Ascomycota sp.* | 234 | 3 | 237 |
| *Exophiala equina* | 151 | 74 | 225 |
| *Gibberella zeae* | 116 | 103 | 219 |
| *Malassezia restricta* | 28 | 189 | 217 |
| *Trametes versicolor* | 94 | 100 | 194 |
| *Ascochyta sp.* | 41 | 149 | 190 |
| *Mycosphaerella tassiana* | 77 | 107 | 184 |
| *Cladosporium flabelliforme* | 42 | 122 | 164 |
| *Humicola grisea* | 32 | 114 | 146 |
| *Vishniacozyma carnescens* | 77 | 67 | 144 |
| *Aureobasidium pullulans* | 49 | 88 | 137 |
| *Cyberlindnera fabianii* | 56 | 61 | 117 |
| *Didymellaceae sp.* | 32 | 43 | 75 |
| *Candida albicans* | 17 | 47 | 64 |
| *Fusicolla aquaeductuum* | 1 | 60 | 61 |
| *Kazachstania humilis* | 44 | 8 | 52 |
| *Thanatephorus cucumeris* | 3 | 47 | 50 |
