# Supplementary material for: Fungal Dysbiosis and Intestinal Inflammation in Children With Beta-Cell Autoimmunity
Source: Front Immunol. 2020 Mar 19;11:468. doi: 10.3389/fimmu.2020.00468 (PMC7103650; doi:10.3389/fimmu.2020.00468)
Supplement: Supplementary Table 2 — Summary of the permutational multivariate analysis of variance (PERMANOVA). PERMANOVA statistical tests were performed on the weighted UniFrac distances and Bray Curtis dissimilarity between the (1) fungal and (2) bacterial gut microbial communities profiles of children at risk of the type 1 diabetes development. Statistical tests were run using the adonis function in R package vegan (with 999 permutations). The significant (<0.05) values shown in bold. [file Table_2.DOCX]

**Supplementary Table 2. Summary of the permutational multivariate analysis of variance (PERMANOVA).** PERMANOVA statistical tests were performed on the weighted UniFrac distances and Bray Curtis dissimilarity between the (1) fungal and (2) bacterial gut microbial communities profiles of children at risk of the type 1 diabetes development. Statistical tests were run using the *adonis* function in R package vegan (with 999 permutations). The significant (<0.05) values shown in bold.

|  | **weighted UniFrac** | | | | **Bray Curtis** | | |
| --- | --- | --- | --- | --- | --- | --- | --- |
| **FUNGAL COMMUNITY** | **Variable** | ***F*** | ***R²*** | **p-value** | ***F*** | ***R²*** | **p-value** |
|  | Age | 0.226 | 0.005 | 0.949 | 0.451 | 0.009 | 0.938 |
|  | Gender | 0.428 | 0.008 | 0.726 | 1.120 | 0.022 | 0.273 |
|  | HLA risk class | 1.635 | 0.032 | 0.171 | 1.191 | 0.023 | 0.253 |
|  | AAb positivity | 0.952 | 0.019 | 0.334 | 1.168 | 0.023 | 0.271 |
|  | Number Of Aabs | 0.788 | 0.063 | 0.617 | 1.231 | 0.095 | 0.197 |
|  | T1D diagnosis | 0.666 | 0.013 | 0.499 | 1.518 | 0.029 | 0.139 |
|  | **Cluster** | 3.968 | 0.301 | **0.001** | 3.119 | 0.253 | **0.001** |
|  | ***Saccharomyces*** | 92.098 | 0.648 | **0.001** | 20.716 | 0.293 | **0.001** |
|  | ***Candida*** | 14.420 | 0.224 | **0.001** | 15.223 | 0.233 | **0.001** |
|  | **weighted UniFrac** | | | | **Bray Curtis** | | |
| **BACTERIAL COMMUNITY** | **Variable** | ***F*** | ***R²*** | **p-value** | ***F*** | ***R²*** | **p-value** |
|  | **Age** | 4.479 | 0.082 | **0.015** | 2.671 | 0.051 | **0.001** |
|  | Gender | 1.103 | 0.022 | 0.293 | 1.019 | 0.020 | 0.428 |
|  | **HLA risk class** | 8.266 | 0.142 | **0.001** | 2.229 | 0.043 | **0.007** |
|  | **AAb positivity** | 3.115 | 0.059 | **0.039** | 1.090 | 0.021 | 0.331 |
|  | Number Of Aabs | 1.550 | 0.117 | 0.129 | 1.057 | 0.083 | 0.324 |
|  | T1D diagnosis | 0.875 | 0.017 | 0.398 | 1.328 | 0.026 | 0.125 |
|  | **Cluster** | 7.459 | 0.448 | **0.001** | 1.683 | 0.155 | **0.002** |
|  | ***Clostridiales*** | 81.447 | 0.620 | **0.001** | 7.890 | 0.136 | **0.001** |
|  | ***Bacteroidales*** | 85.912 | 0.632 | **0.001** | 8.083 | 0.139 | **0.001** |
