# Supplementary material for: Fungal Dysbiosis and Intestinal Inflammation in Children With Beta-Cell Autoimmunity
Source: Front Immunol. 2020 Mar 19;11:468. doi: 10.3389/fimmu.2020.00468 (PMC7103650; doi:10.3389/fimmu.2020.00468)
Supplement: Supplementary Table 3 — Summary of the analysis of similarities (ANOSIM). ANOSIM statistical tests were performed on the weighted UniFrac distances and Bray Curtis dissimilarity between the (1) fungal and (2) bacterial gut microbial communities profiles of children at risk of the type 1 diabetes development. Statistical tests were run using the anosim function in R package vegan (with 999 permutations). The Benjamini-Hochberg False Discovery Rate (FDR) correction for multiple testing was used to calculate q-values. The significant (<0.05) values shown in bold. [file Table_3.DOCX]

**Supplementary Table 3. Summary of the analysis of similarities (ANOSIM).** ANOSIM statistical tests were performed on the weighted UniFrac distances and Bray Curtis dissimilarity between the (1) fungal and (2) bacterial gut microbial communities profiles of children at risk of the type 1 diabetes development. Statistical tests were run using the *anosim* function in R package vegan (with 999 permutations). The Benjamini-Hochberg False Discovery Rate (FDR) correction for multiple testing was used to calculate q-values. The significant (<0.05) values shown in bold.

|  | **weighted UniFrac** | | | | | | **Bray Curtis** | | |
| --- | --- | --- | --- | --- | --- | --- | --- | --- | --- |
| **FUNGAL COMMUNITY** | **Comparison** | | ***R*** | **p-value** | | **q-value** | ***R*** | **p-value** | **q-value** |
|  |  |  |  |  |  |  |  |  |  |
|  | Cluster 5 | Cluster 4 | 0.067 | 0.139 | | 0.167 | 0.0111 | 0.39 | 0.39 |
|  |  |  |  |  |  |  |  |  |  |
|  | Cluster 5 | Cluster 1 | 0.596 | **0.001** | | **0.003** | 0.53 | **0.002** | **0.009** |
|  |  |  |  |  |  |  |  |  |  |
|  | Cluster 4 | Cluster 1 | 0.111 | **0.023** | | **0.046** | 0.053 | 0.08 | 0.16 |
|  |  |  |  |  |  |  |  |  |  |
|  | **weighted UniFrac** | | | | | | **Bray Curtis** | | |
| **BACTERIAL COMMUNITY** | **Comparison** | | ***R*** | **p-value** | | **q-value** | ***R*** | **p-value** | **q-value** |
|  |  |  |  |  |  |  |  |  |  |
|  | Cluster 5 | Cluster 4 | -0.027 | | 0.56 | 0.56 | -0.096 | 0.84 | 0.84 |
|  |  |  |  |  |  |  |  |  |  |
|  | Cluster 5 | Cluster 1 | 0.76 | **0.001** | | **0.003** | 0.36 | **0.001** | **0.003** |
|  |  |  |  |  |  |  |  |  |  |
|  | Cluster 4 | Cluster 1 | 0.52 | **0.001** | | **0.003** | 0.26 | **0.001** | **0.003** |
|  |  |  |  |  |  |  |  |  |  |
|  | | | | | | | | | |
